# Diagnostic performance of a rapid whole blood-based RT-LAMP method for malaria diagnosis among apparently healthy blood donors and febrile neonates in Cameroon

**DOI:** 10.1371/journal.pone.0246205

**Published:** 2021-01-28

**Authors:** Sylvie Georgette Zebaze Temgoua Kemleu, Laure Ngando, Elvige Nguekeng, Balotin Fogang, Marie Mafo Kapen, Styve Iruch Fopa, Marie Florence Biabi, Estelle Essangui, Jules Clement Assob Nguedia, Lawrence Ayong

**Affiliations:** 1 Malaria Research Unit, Centre Pasteur du Cameroun, Yaounde, Cameroon; 2 Department of Medical Laboratory Sciences, Faculty of Health Sciences, University of Buea, Buea, Cameroon; 3 Bacteriology Unit, Centre Pasteur du Cameroun, Yaounde, Cameroon; 4 University Teaching Hospital (CHU), Yaounde, Cameroon; 5 Faculty of Medicine and Pharmaceutical Sciences, University of Douala, Douala, Cameroon; Instituto de Salud Global de Barcelona, SPAIN

## Abstract

Light microscopy and rapid diagnostic tests are the two commonly used methods for malaria diagnosis that rely on the direct use of unprocessed blood samples. However, both methods do not have the level of sensitivity required for malaria diagnosis in cases of low density parasitaemia. We report here the diagnostic performance of a whole blood-based reverse transcription loop-mediated isothermal amplification method for *Plasmodium falciparum* malaria diagnosis in apparently healthy blood donors and febrile neonates in Cameroon. The presence of malaria parasites in whole blood samples was determined by light microscopy, antigen-based rapid diagnostic test (RDT), and by RT-LAMP using a “lyse and amplify” experimental protocol. Of the 256 blood donors tested, 36 (14.1%) were positive for malaria parasites by light microscopy, 38 (14.8%) were positive by RDT whereas 78 (30.5%) were positive by RT-LAMP. Only light microscopy and RT-LAMP detected infection among the febrile neonates (279 neonates, median age: 2 days, range: 1–9 days), with positivity rates of 8.6% and 12.2%, respectively. The overall concordance between the three methods were 75.9% for RT-LAMP and light microscopy, 75.1% for RT-LAMP and RDT, and 83.9% for light microscopy and RDT. Blood parasite densities were significantly lower in the neonates (mean: 97.6, range: 61–192 parasites/μL) compared to the blood donors (mean: 447.8, range: 63–11 000 parasites/μL). Together, the study demonstrates the usefulness of whole blood RT-LAMP for use in rapid pre-screening of blood donors and suspected neonates to avert severe consequences of *P*. *falciparum* infections.

## Introduction

Malaria is a vector borne disease caused by protozoan parasites of the genus *Plasmodium*. Vector transmission of malaria parasites that occurs during the blood meal of an infected female anopheles mosquito is the most common mode of transmission [1, 2]. However, other modes of transmission exist, including vertical transmission from infected mothers to their unborn babies (congenital malaria) [3–6], and horizontal transmission from infected blood donors to their recipients (transfusion-transmitted malaria) [7–9]. In fact, both congenital and transfusion-transmitted malarias are common and represent significant public health problems, particularly in areas with high stable transmission of *Plasmodium falciparum* [5, 7, 9, 10]. In either case, the most effective preventive measure is the proactive testing and treatment of infected blood donors and pregnant women, thereby preventing onward transmission of the parasite. Alternatively, early diagnosis and prompt treatment of blood recipients or infected neonates is the most effective strategy for averting serious complication of the disease in such vulnerable groups. Diagnosing malaria in asymptomatic blood donors or pregnant women, as well as early diagnosis in non-immune babies and blood recipients is challenging due to the extremely low parasite densities that characterize such patient groups [11–13]. In addition, it is pertinent to note that most cases of neonate malaria are misdiagnosed initially because of limited awareness about congenital malaria, lack of user-friendly and high sensitivity tests capable of detecting low level infections in such patient groups, and the generally non-specific nature of early-stage malaria symptoms.

Traditionally, malaria diagnosis is established by the microscopic detection of parasites on Giemsa-stained thick or thin blood smears, or using one of several antigen-based immunochromatographic methods otherwise known as rapid diagnostic tests (RDTs) [14]. Although considered the “gold standard” for malaria diagnosis in hyper-endemic countries, microscopy-based methods for malaria diagnosis are highly unreliable, particularly in patients with low density parasitaemia (<50 parasites/ μl of blood) [15, 16]. Additionally, the method is time-consuming and labor-intensive [16]. Similarly, immunochromatographic methods, although user-friendly, are not sensitive enough for use in point-of-need diagnosis of low-density malaria parasitaemia (sensitivity limits > 100 parasites/μl of blood) [17].

The most reliable approach for diagnosing low-density parasitaemia is by use of molecular methods such as PCR and loop-mediated isothermal nucleic acid amplification methods [14]. Indeed, recent advances in PCR-based methods allow the detection of less than 1 parasite/μl of blood [18, 19], making the method highly appropriate for use in cases of low level parasitaemia. However, PCR-based methods are expensive and laborious, requiring a purification step to eliminate polymerase inhibitors that are commonly found in body fluids [13, 20, 21]. An alternative but more recent molecular approach for malaria diagnosis is the loop-mediated isothermal amplification (LAMP) that may be employed on purified DNA or RNA [22, 23], or directly on whole blood samples [24]. At least two LAMP-based methods for malaria diagnosis have been reported, with sensitivity limits in the range of (0.2–0.08) and turnaround times similar to that of rapid diagnostic tests procedure [14, 24]. We aim in this study to compare the diagnostic performances of a recently developed RT-LAMP method for malaria [24], with routine microscopy and RDT in the detection of *P*. *falciparum* amongst asymptomatic blood donors and febrile neonates in a hyper-endemic zone in Cameroon.

## Methods

### Study design and participants

A cross-sectional study was conducted at the Neonatology Unit of the Yaounde Central Hospital from June 2018 to November 2018 to assess the prevalence of congenital malaria in the city of Yaounde, and at the Blood Bank Unit of the Yaounde University Teaching Hospital from July 2019 to September 2019 to determine the frequency of malaria parasite contamination of transfusable blood products in the area. The inclusion criteria were febrile neonates of age <10 days reporting to nearby health facilities for medical consultation, and healthy looking adult blood donors. Written informed consents were obtained from participants or their legal representatives.

### Ethical consideration

Ethical approval of the study was obtained from the Committee on Human Subjects Research of the Catholic University of Central Africa in Yaounde, Cameroon (protocol number 2018/0698/CEIRSH/ESS/MIM). Administrative approvals were obtained from heads of the participating health facilities.

### Sample collection and laboratory analyses

A non-random sampling technique was used to select the study participants. Approximately 60 μl of leftover blood samples, collected by hospital staff for routine medical analysis or destined for the blood bank during the study period, were obtained at each collection site. Thick blood films (5 μl of whole blood) were prepared and stained using 10% Giemsa solution for 15 minutes. The films were analyzed by light microscopy (Leica DM750, Tokyo) using a 100X oil immersion objective. Slides were considered negative if parasites were undetected after a count of 1000 leucocytes. Parasitaemia was determined by assuming a leucocyte count of 8000 white blood cells/μl of blood. All slides were analyzed by at least by two microscopists. In case of a discordant result, a third reading was done by an independent expert microscopist.

The Malaria Ag Pf/pan RDT kit was used for *P*. *falciparum*-specific antigen detection, following the manufacturer’s instructions (Standard Diagnostic Inc., South Korea). Infection with *P*. *falciparum* was also detected by RT-LAMP using the whole blood protocol as previously described [24]. Briefly, blood samples were diluted (1:50) in a lysis buffer comprising 10 mM Tris-buffered saline, pH 7.4, 0.2% Triton X-100, and 0.1% bovine serum albumin and kept standing at room temperature for 2 minutes. The lysate (2.5 uL) were immediately added to a reaction mix comprising 2 μl of pre-mixed primers, 15 μL of reconstituted enzyme (ISO-DR001, OptiGene, UK) and 5.5 μl of DEPC-treated water. The RT-LAMP reaction was then realized at 68°C for 45 minutes using a Genie II real-time amplifier. An RT-LAMP inactivation/annealing step of 98–70°C with ramp at 0.1°C per minute was included, and the derived melting curves used to verify the reaction specificity. Samples were considered positive if resulting in an amplification peak with a characteristic annealing temperature of ~86.5°C.

### Data analysis

Data were entered into Microsoft Office Excel and analyzed using Graphpad and an online statistical tool (https://www.medcalc.org/calc/diagnostic_test.php) for descriptive statistics that comprised test positivity rates, accuracy, relative sensitivities and specificities. Differences between diagnostic test methods were determined using the McNemar’s test for paired data, considering P values less than 0.05 as statistically significant.

## Results

### Proportions of *P*. *falciparum* infections by diagnostic method

A total of 279 neonates (median age: 2 days, range: 1–9 days, males: 54.5%) reporting with a fever for consultation in various health facilities in Yaounde, and a total of 256 volunteer blood donors (median age; 25 years, range; 19–45 years, males: 73.3%) were screened for *P*. *falciparum* infection by light microscopy, RDT and whole blood-based RT-LAMP. Of the 279 neonates tested, 24 (8.6%) were positive by light microscopy, 34 (12.2%) were positive by RT-LAMP and none (0%) were positive by RDT. Similarly, 14.1% of the 256 blood donors were positive for *P*. *falciparum* infection by light microscopy, 30.5% were positive by RT-LAMP and 14.8% by RDT. The mean parasite densities in the light microscopy positive samples were 97.6 (range: 61–192) parasites/μl of blood in neonates and 447.8 (range: 63–11 000) parasites/μl in blood donors. Overall, the malaria positivity rates in the two study groups combined were 11.2% for light microscopy, 21.1% for RT-LAMP and 7.1% for RDT. Additionally, the proportion of submicroscopic infections detected by RT-LAMP or RDT, were 17.0% and 6.0%, respectively, indicating a better performance of RT-LAMP as a screening test for *P*. *falciparum* infections in such population groups.

### Concordance and diagnostic differences between light microscopy, RT-LAMP and RDT

Of the 535 neonates and blood donors tested by all three methods, 406 produced similar results (22 positives and 384 negatives) between light microscopy and RT-LAMP, resulting in a calculated concordance of 75.9% between the two methods. The concordance between light microscopy and RDT was 83.9%, whereas the concordance between RDT and RT-LAMP was 75.1%. Of the discordant pairs (Table 1), 70.5% (91/129) were positive by RT-LAMP but negative by light microscopy (P<0.0001), 78.2% (104/133) were positive by RT-LAMP but negative by RDT (P<0.0001), and 62.8% were positive by light microscopy but negative by RDT (P = 0.02). Together, these findings show an overall good agreement between the various methods, and a better performance of RT-LAMP over light microscopy and RDT in terms of test positivity rates.

**Table 1:** Proportion of discordant pairs between test methods.

Negative	LM	RT-LAMP	RDT
Positive			
**LM**	-	38 (29.5%)	54 (62.8%)
**RT-LAMP**	91 (70.5%)	-	104 (78.2%)
**RDT**	32 (37.2%)	29 (21.8%)	-

### Relative accuracy, sensitivity and specificity of whole blood RT-LAMP method

As shown in Table 2, the accuracy with which RT-LAMP detected malaria parasite infection was 75.89% (95%CI; 72.03% - 79.45%) when compared to light microscopy, and 75.14% (95%CI: 71.25% - 78.75%) when compared to RDT. The sensitivity of RT-LAMP determined amongst the light microscopy positive samples was 36.7% (95%CI: 24.59% - 50.10%), and 23.7% (95%CI: 11.44% - 40.24%) when determined based on RDT positives. Similarly, the specificity of RT-LAMP as determined amongst the light microscopy negative samples was 80.8% (95%CI: 77.01% - 84.29%), and 79.1% (95%CI: 75.23% - 82.57%) when determined amongst the RDT negative samples. Taken together, the data show high accuracy of RT-LAMP when compared to either light microscopy or RDT, reflecting a high overall probability of correctly classifying *P*. *falciparum* infected neonates and blood donors using the whole blood RT-LAMP method.

**Table 2:** Accuracy, sensitivity and specificity of whole blood RT-LAMP.

Comparator method	RT-LAMP		
	Accuracy [95%CI]	Relative sensitivity [95%CI]	Relative specificity [95%CI]
**Light microscopy**	75.89% [72.03% - 79.45%]	36.7% [24.59% - 50.10%]	80.8% [77.01% - 84.29%]
**RDT**	75.14% [71.25% - 78.75%]	23.7% [11.44% - 40.24%]	79.1% [75.23% - 82.57%]

## Discussion

Accurate and early diagnosis of malaria followed by prompt treatment is the only way to avert serious complications of congenitally transmitted malaria in neonates and transfusion-transmitted malaria. This study was conducted to compare the performance of a recently developed RT-LAMP method with light microscopy and RDT for use in the diagnosis of both congenital and transfusion-transmitted malaria in resource-limited environments.

Interestingly, the whole blood RT-LAMP out-performed both light microscopy and RDT in terms of infection detection rates in both study populations. The proportion of diagnosed malaria cases by RT-LAMP was about twice the proportion of cases by either light microscopy or RDT among the blood donors, and 1.4 times greater than malaria cases by light microscopy in the febrile neonates. This increased proportion of RT-LAMP positives may be attributed to the extremely low detection limit (0.08 parasites/μl whole blood) of the new method [24], and the fact that most of the identified cases (neonates and blood donors) presented with extremely low parasite densities. RDT, with a *P*. *falciparum* detection limit over 100 parasites/μl was negative in all neonate samples tested. The prevalence of malaria in the neonate group (8.6% by light microscopy and 12.2% by RT-LAMP) are similar to recent reports in the South West Region of Cameroon where 14.4% prevalence of congenital malaria by microscopy has been reported [3], and significantly low when compared to earlier reports in Calabar, Nigeria where the prevalence of congenital malaria among neonates with suspected sepsis was 35% [25]. The observed decrease in congenital malaria prevalence in our study population can be attributed to widespread implementation of intermittent malaria preventive measures in the country that include obligatory sulfadoxine-pyrimethamine administration and bed net use among pregnant women as from their first trimester. The obtained prevalence of malaria among the blood donors is similar to recent observations by Okalla *et al*. [10] using light microscopy wherein a prevalence of 14.1% was reported among blood donors in the littoral region of Cameroon.

Despite the high positivity rate of whole blood RT-LAMP when compared to light microscopy and RDT, concordance between the different tests were high (>75%), indicating that all three methods may be used interchangeable in the study population. However, RT-LAMP produced the highest proportion of test positives when comparing the discordant pairs, suggesting that the method is suitable as a screening test for *P*. *falciparum* infection in both study populations. RT-LAMP was highly accurate (>75% accuracy) in identifying light microscopy and RDT confirmed malaria cases. However, despite the high specificity (~80%) when compared to light microscopy or RDT, the sensitivity performances of whole blood RT-LAMP were low (36.7% relative to light microscopy and 23.7% relative to RDT). These observations contrast previous findings by Kemleu *et al*. [24], wherein a sensitivity of 90% was reported when determined among RT-PCR positives. This discrepancy may be due to reduced reliability of both light microscopy and RDT in patients with low density parasitaemia and in recently treated participants.

In conclusion, this study demonstrates the usefulness of whole blood malaria RT-LAMP for use in malaria diagnosis among blood donors and in suspected neonates. Additionally, the study confirms the occurrence of congenital malaria as well as the high prevalence of *P*. *falciparum* infections in blood samples destined for transfusion in the city of Yaounde, Cameroon, thereby requiring policy changes in order to avert serious complications of both congenital and transfusion-transmitted malaria in the country.

## References

[1] Trung HD, Bortel WV, Sochantha T, Keokenchanh K, Quang NT, Cong LD, et al Malaria transmission and major malaria vectors in different geographical areas of Southeast Asia. Trop Med Int Health. 2004;9: 230–237. 10.1046/j.1365-3156.2003.01179.x 15040560

[2] World Health Organization. World malaria report 2019. WHO; 2019. Report No.: ISBN 978-92-4-156572-1.

[3] Tatiana NMP, Amin ET, Njumkeng C, Fondungallah AJ, Peter NF. Congenital Malaria: Prevalence and Risk Factors in the Fako Division of the Southwest Region of Cameroon.: 8.

[4] Moya-Alvarez V, Abellana R, Cot M. Pregnancy-associated malaria and malaria in infants: an old problem with present consequences. Malar J. 2014;13: 271 10.1186/1475-2875-13-271 25015559 PMC4113781

[5] Ja B, Ee M, A A-N, I A. Global prevalence of congenital malaria: A systematic review and meta-analysis. Eur J Obstet Gynecol Reprod Biol. 2020 [cited 24 Aug 2020]. 10.1016/j.ejogrb.2020.06.025 32620512

[6] Omer SA, Adam I, Noureldien A, Elhaj H, Guerrero-Latorre L, Silgado A, et al Congenital Malaria in Newborns Delivered to Mothers with Malaria-Infected Placenta in Blue Nile State, Sudan. J Trop Pediatr. 2020;66: 428–434. 10.1093/tropej/fmz083 31951264

[7] Verra F, Angheben A, Martello E, Giorli G, Perandin F, Bisoffi Z. A systematic review of transfusion-transmitted malaria in non-endemic areas. Malar J. 2018;17 10.1186/s12936-018-2181-0 29338786 PMC5771189

[8] Antwi-Baffour S, Kyeremeh R, Amoako AP, Annison L, Tetteh JO-M, Seidu MA. The Incidence of Malaria Parasites in Screened Donor Blood for Transfusion. Malar Res Treat. 2019;2019 10.1155/2019/1457406 31885856 PMC6899260

[9] Wen-Ming H, Jian-Feng C, Xiang Z, Yi-Lin H. [Epidemiological investigation of blood transfusion—induced malaria caused by an imported case with Plasmodium falciparum infection]. Zhongguo Xue Xi Chong Bing Fang Zhi Za Zhi Chin J Schistosomiasis Control. 2019;31: 439–440. 10.16250/j.32.1374.2018254 31612685

[10] Okalla Ebongue C, Ngouadjeu Dongho E, Texier G, Nda Mefo’o J-P, Sume Etapelong G, Ayong L, et al Residual risk of transfusion-transmitted malaria infection in a malaria endemic sub-Saharan African setting. Transl Med Commun. 2017;2: 4 10.1186/s41231-017-0013-9

[11] Romani L, Pane S, Severini C, Menegon M, Foglietta G, Bernardi S, et al Challenging diagnosis of congenital malaria in non-endemic areas. Malar J. 2018;17 10.1186/s12936-018-2614-9 30551740 PMC6295090

[12] D’Alessandro U, Olaleye BO, McGuire W, Langerock P, Bennett S, Aikins MK, et al Mortality and morbidity from malaria in Gambian children after introduction of an impregnated bednet programme. Lancet Lond Engl. 1995;345: 479–483. 10.1016/s0140-6736(95)90582-0 7861874

[13] Moody A. Rapid diagnostic tests for malaria parasites. Clin Microbiol Rev. 2002;15: 66–78. 10.1128/cmr.15.1.66-78.2002 11781267 PMC118060

[14] Ayong L, Moukoko CEE, Mbacham WF. Diagnosing Malaria: Methods, Tools, and Field Applicability. Methods Mol Biol Clifton NJ. 2019;2013: 73–82. 10.1007/978-1-4939-9550-9_5 31267494

[15] Tebit KE, Njunda LA, Assob JCN, Kamga HLF, Nsagha SD, Mokenyu MD. Comparison of capillary and venous blood using blood film microscopy in the detection of malaria parasites: A hospital based study. 2013 10.1097/QAD.0b013e3283610ec7 23727942 PMC4494684

[16] Noubouossie D, Tagny CT, Same-Ekobo A, Mbanya D. Asymptomatic carriage of malaria parasites in blood donors in Yaoundé. Transfus Med. 2012;22: 63–67. 10.1111/j.1365-3148.2011.01121.x 22141368

[17] Bourgeois N, Boutet A, Bousquet P-J, Basset D, Douard-Enault C, Charachon S, et al Comparison of three real-time PCR methods with blood smears and rapid diagnostic test in Plasmodium sp. infection. Clin Microbiol Infect. 2010;16: 1305–1311. 10.1111/j.1469-0691.2009.02933.x 19840032

[18] Imwong M, Hanchana S, Malleret B, Rénia L, Day NPJ, Dondorp A, et al High-throughput ultrasensitive molecular techniques for quantifying low-density malaria parasitemias. J Clin Microbiol. 2014;52: 3303–3309. 10.1128/JCM.01057-14 24989601 PMC4313154

[19] Adams M, Joshi SN, Mbambo G, Mu AZ, Roemmich SM, Shrestha B, et al An ultrasensitive reverse transcription polymerase chain reaction assay to detect asymptomatic low-density Plasmodium falciparum and Plasmodium vivax infections in small volume blood samples. Malar J. 2015;14: 520 10.1186/s12936-015-1038-z 26701778 PMC4690410

[20] Stresman G, Kobayashi T, Kamanga A, Thuma PE, Mharakurwa S, Moss WJ, et al Malaria research challenges in low prevalence settings. Malar J. 2012;11: 353 10.1186/1475-2875-11-353 23098277 PMC3502576

[21] Wongsrichanalai C, Barcus MJ, Muth S, Sutamihardja A, Wernsdorfer WH. A Review of Malaria Diagnostic Tools: Microscopy and Rapid Diagnostic Test (RDT). American Society of Tropical Medicine and Hygiene; 2007 Available: https://www.ncbi.nlm.nih.gov/books/NBK1695/ 18165483

[22] Notomi T, Okayama H, Masubuchi H, Yonekawa T, Watanabe K, Amino N, et al Loop-mediated isothermal amplification of DNA. Nucleic Acids Res. 2000;28: E63 10.1093/nar/28.12.e63 10871386 PMC102748

[23] Chander Y, Koelbl J, Puckett J, Moser MJ, Klingele AJ, Liles MR, et al A novel thermostable polymerase for RNA and DNA loop-mediated isothermal amplification (LAMP). Front Microbiol. 2014;5 10.3389/fmicb.2014.00395 25136338 PMC4117986

[24] Kemleu S, Guelig D, Eboumbou Moukoko C, Essangui E, Diesburg S, Mouliom A, et al A Field-Tailored Reverse Transcription Loop-Mediated Isothermal Assay for High Sensitivity Detection of Plasmodium falciparum Infections. PLoS ONE. 2016;11 10.1371/journal.pone.0165506 27824866 PMC5100904

[25] Mogtomo MLK, Foko LPK, Okoubalimba EVA, Enyegue EE, Ngane ARN. High Risk of Transfusion-Transmitted Malaria (TTM) from Student Blood Donors Living in the Town of Douala, Cameroon. J Clin Infect Dis Pract. 2016;1: 1–5. 10.4172/2476-213X.1000108

